# Dataset comprising pesticides, pharmaceuticals and personal care products, and industrial chemicals detected in streams and rivers of Central Chile

**DOI:** 10.1016/j.dib.2023.109600

**Published:** 2023-09-20

**Authors:** Pedro A． Inostroza, Sebastian Elgueta, Melis Muz, Martin Krauss, Werner Brack, Thomas Backhaus

**Affiliations:** a Department of Biological and Environmental Sciences, University of Gothenburg, Gothenburg, Sweden; b Núcleo en Ciencias Ambientales y Alimentarias (NCAA), Universidad de las Américas, Chile; c Facultad de Medicina Veterinaria y Agronomía, Universidad de las Américas, Sede Providencia, Chile; d Department of Effect-Directed Analysis, Helmholtz Centre for Environmental Research – UFZ, Leipzig, Germany; e Department of Evolutionary Ecology and Environmental Toxicology, Goethe University Frankfurt/Main, Frankfurt/Main, Germany; f Institute for Environmental Research, RWTH Aachen University, Aachen, Germany

**Keywords:** Target screening, Chemicals of emerging concern, Surface waters, River pollution, LVSPE

## Abstract

Chemical pollution caused by synthetic organic chemicals at low concentrations in the environment poses a growing threat to the ecological status of aquatic ecosystems. These chemicals are regularly released into surface waters through both treated and untreated effluents from wastewater treatment plants (WWTPs), agricultural runoff, and industrial discharges. Consequently, they accumulate in surface waters, distribute amongst environmental compartments according to their physicochemical properties, and cause adverse effects on aquatic organisms. Unfortunately, there is a lack of data regarding the occurrence of synthetic organic chemicals, henceforth micropollutants, in South American freshwater ecosystems, especially in Chile.

To address this research gap, we present a comprehensive dataset comprising concentrations of 153 emerging chemicals, including pesticides, pharmaceutical and personal care products (PPCPs), surfactants, and industrial chemicals. These chemicals were found to co-occur in surface waters within Central Chile, specifically in the River Aconcagua Basin. Our sampling strategy involved collecting surface water samples from streams and rivers with diverse land uses, such as agriculture, urban areas, and natural reserves. For sample extraction, we employed an on-site large-volume solid phase extraction (LVSPE) device. The resulting environmental extracts were then subjected to wide-scope chemical target screening using gas chromatography and liquid chromatography high-resolution mass spectrometry (GC- and LC—HRMS).

The dataset we present holds significant value in assessing the chemical status of water bodies. It enables comparative analysis of pollution fingerprints associated with emerging chemicals across different freshwater systems. Moreover, the data can be reused for environmental risk assessment studies. Its utilisation will contribute to a better understanding of the impact and extent of chemical pollution in aquatic ecosystems, facilitating the development of effective mitigation strategies.

Specifications Table

**Table:** 

Subject	Pollution
Specific subject area	Synthetic organic pollutants occurring in surface running waters
data format	analysed, filtered
Type of data	Table
Data collection	The data were acquired via gas and liquid chromatography-high-resolution mass spectrometry. Target screening was conducted for 861 chemicals using an UltiMate 3000 LC system (Thermo Scientific, Germany) coupled to a quadrupole-Orbitrap MS (Q Exactive Plus, Thermo Scientific, Germany) with a heated electrospray ionization (ESI) source. A retrospective analysis was applied to 150 of the 861 target chemicals. More hydrophobic analytes, comprised of 36 chemicals, were re-evaluated using a TRACE 1310 GC system (Thermo Scientific, Germany) coupled to a quadrupole-Orbitrap MS (Q Exactive, Thermo Scientific, Germany) equipped with a Thermal Desorption Unit (TDU-2; Gerstel, Mülheim, Germany) and a cooled injection system (CIS; Gerstel).
Data source location	Data were stored: University of Gothenburg and the Helmholtz Centre for Environmental Research – UFZ City/Town/Region: Gothenburg, Västra Götaland and Leipzig, Saxony Country: Sweden and Germany Data were collected: Latitude and longitude (WGS84): RS1 (−32.854358N −70.390044 E), RS2 (−32.509769N −70.452537 E), RS3 (−33.003752N −71.126355 E), T1 (−32.765909N −70.613844 E), T2 (−32.695661N −71.212179 E), T3 (−32.938952N −71.329491 E), R1 (−32.852416N −70.502894 E), R2 (−32.762305N −70.839624 E), and R3 (−32.916946N −71.425322 E).
Data accessibility	Repository name: zenodo Data identification number: 10.5281/zenodo.8088841 Direct URL to data: https://zenodo.org/record/8088841

## 1 Value of the Data

• Environmental concentrations of emerging chemicals are valuable in defining the pollution status of aquatic environments and conducting studies on environmental risk assessment.
• The introduced dataset can aid in subsequent studies involving prioritisation analysis and the establishment of environmental quality standards.
• The reported information can be utilised by researchers and local authorities to facilitate further pollution management efforts.
• The dataset provides a comprehensive overview of pesticides, pharmaceuticals and personal care products, surfactants, and industrial chemicals in surface water in central Chile.
• The data can be used by risk assessors to propose mitigation strategies and surveillance systems.

## 2 Data Description

The dataset in this study originates from surface-running water samples collected from nine distinct sampling sites within the River Aconcagua basin. The dataset is reported in tabular format and is available in both Rdata (RDS) and tab-separated values (TSV) formats. The dataset can be accessed at [1]. For each reported substance, the dataset includes essential identifiers such as the CAS Registry Number (CAS RN), the International Chemical Identifier (InChI), its hashed InChIKey counterpart, and the Simplified Molecular Input Line Entry System (SMILES) identifiers. To complement the dataset, the mode of action (MoA) information of each chemical was retrieved from Busch et al. [2] and gaps were filled through searching in the Elsevier Bibliographic Database (Scopus) and Google Scholar as described in Inostroza et al. [3]. The RDS and TSV files contain the columns defined in Table 1, while an overview of detected and quantified micropollutants is provided in Table 2.

**Table 1:** Data description.

Columns	Description
chemical_name	Name of the emerging chemical
cas_number	CAS Registry Number use as chemical identifier
InChIKey	Textual identifier for chemical substances
SMILES	Line notation for chemical structure
DTXSID	Distributed Structure-Searchable Toxicity substance identifier
type	Type of chemical (parent or transformation product)
main_source	Urban areas, agriculture derived chemical, or multiple sources
class_1	Use category (e.g., pharmaceutical, pesticide, biocide, etc.)
class_2	Sub-use category (e.g., antibiotic, herbicide, plasticizer, etc.)
class_3	Sub-use category (e.g., benzodiazepine, organophosphate, etc.)
alternative_class	Known alternative use (e.g., veterinary pharmaceutical, etc.)
MoA	Mechanism of action of the chemical
MDL	Method detection limit in ng/L
RS1	Concentration at RS1 in ng/L
RS2	Concentration at RS2 in ng/L
RS3	Concentration at RS3 in ng/L
T1	Concentration at T1 in ng/L
T2	Concentration at T2 in ng/L
T3	Concentration at T3 in ng/L
R1	Concentration at R1 in ng/L
R2	Concentration at R2 in ng/L
R3	Concentration at R3 in ng/L

**Table 2:** Summary of data provided.

	RS1	RS2	RS3	T1	T2	T3	R1	R2	R3
Detected micropollutants	28	18	18	80	46	79	39	46	71
Quantified micropollutants	19	9	9	69	33	64	25	28	56

## 3 Experimental Design, Materials and Methods

### 3.1 Sampling design and water collection

**Fig. 1:**
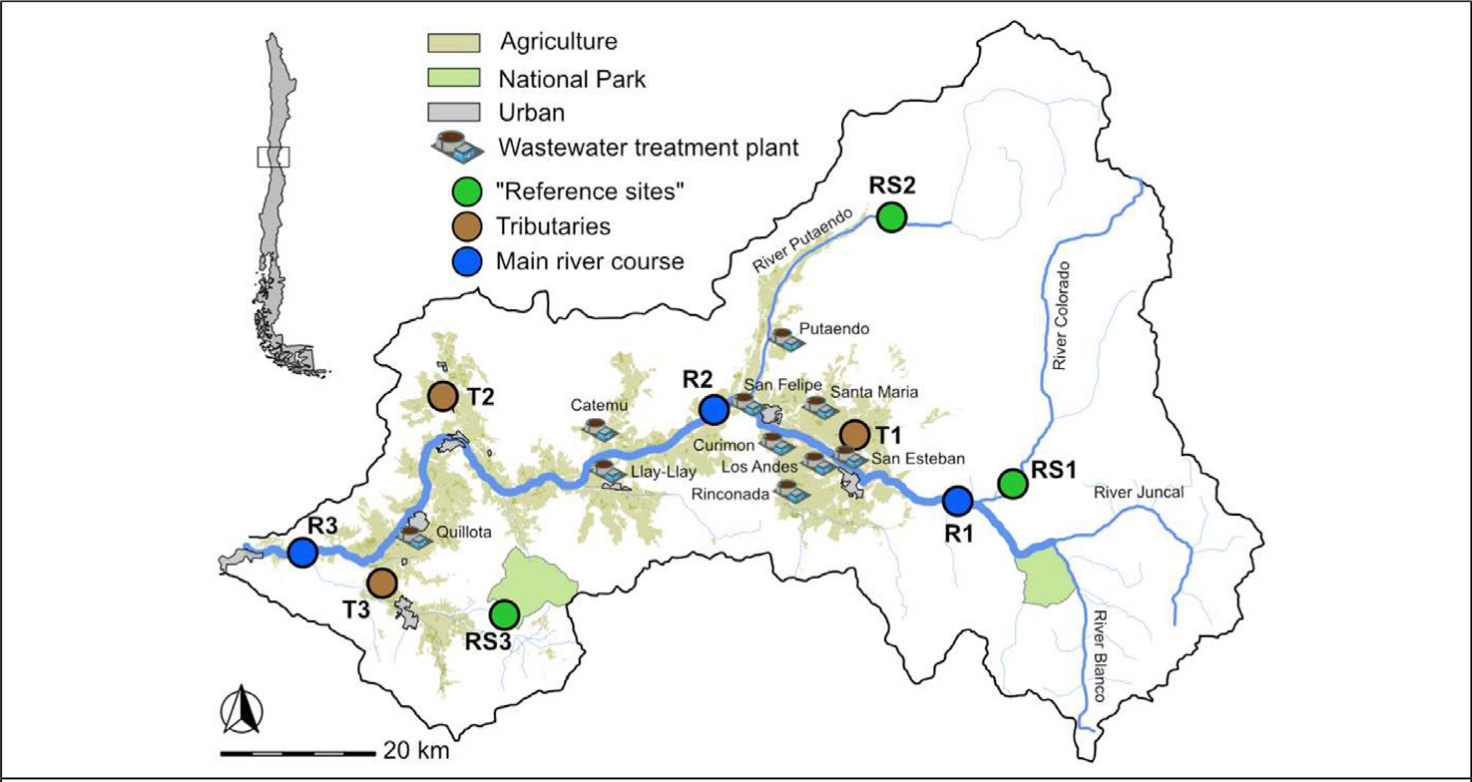
Location of sampling sites through the River Aconcagua Basin. Sites featuring “low” urban/agriculture pressures (“Reference sites”) in green, sites running through agricultural areas in brown, and sites from the main course of the river in light blue. Selected land use (agriculture, urban areas, and national parks) and location of WWTPs are showed in the figure. (For interpretation of the references to colour in this figure legend, the reader is referred to the web version of this article.)

Surface water samples were collected in nine sampling sites in October 2018 (dry season). Sampling sites were selected based on the type of land use. Three sampling sites were characterised by low urban/agriculture pressures (RS1 and RS2 in middle mountain areas and RS3 within a national park) and were designated as “reference sites”. Three sites were located at small tributary streams running throughout agricultural areas (T1 and T3) and through mixed land uses (agricultural areas and small urban areas (T2)). Lastly, three sites were part of the main course of the River Aconcagua (R1, R2, and R3; Fig. 1). Moreover, ten middle-sized WWTPs, featuring aeration ponds and activated sludge technologies, are located across the basin, serving about 405,000 residents. However, only five of them discharge directly into the main course of the River Aconcagua and the others discharge into its tributaries. Further details regarding the sampling sites can be found in Table 3 and geographic coordinates of the WWTPs in Table 4.

**Table 3:** Additional sampling site information. Geographic coordinates in decimal degree (WGS84).

Site ID	Type	Latitude (N)	Longitude (E)	Odour	Colour	Floating material
RS1	“Reference”	−32.854358	−70.390044	none	colourless	none
RS2	“Reference”	−32.509769	−70.452537	none	colourless	none
RS3	“Reference”	−33.003752	−71.126355	none	colourless	none
T1	Tributary	−32.765909	−70.613844	farm	grey/brown	organic material
T2	Tributary	−32.695661	−71.212179	sewage	colourless	debris
T3	Tributary	−32.938952	−71.329491	sewage	colourless	organic material, bloom
R1	Main river	−32.852416	−70.502894	none	grey	foam
R2	Main river	−32.762305	−70.839624	none	colourless	foam
R3	Main river	−32.916946	−71.425322	none	colourless	organic material

**Table 4:** Location of the WWTPs discharging within the River Aconcagua basin. Geographic coordinates in decimal degree (WGS84).

WWTP name	Latitude (N)	Longitude (E)	Receiving water body
Llay-Llay	−32.841182	−70.986937	Stream Los Loros
San Esteban	−32.812008	−70.608260	River Aconcagua
Putaendo	−32.636504	−70.721839	River Putaendo
Santa Maria	−32.740647	−70.664266	Stream San Francisco
Curimon	−32.772475	−70.708835	River Aconcagua
Rinconada	−32.833140	−70.706487	Stream Pocuro
Los Andes	−32.806957	−70.633012	River Aconcagua
San Felipe	−32.748100	−70.740988	River Aconcagua
Quillota	−32.903656	−71.278717	River Aconcagua
Catemu	−32.781818	−70.972715	Stream Catemu

For the collection of surface water samples, an on-site large-volume solid phase extraction (LVSPE) device (MAXX Mess-und Probenahmetechnik GmbH, Rangendingen, Germany) was employed. This device allowed the collection of large volume of water samples. A detailed description of the LVSPE sampler, method development, and extraction recoveries can be found in Schulze et al. [4]. Cartridge preparation, conditioning, and extraction is also explained in detail in Schulze et al. [4]. Subsequent liquid chromatography (LC) and gas chromatography (GC) analyses followed the analytical protocols outlined by Nanusha et al. [5] and Machate et al. [6]. To ensure sample integrity, the remaining extracts were stored at −20 °C for future bioassays.

### 3.2 Target chemical screening

Micropollutant monitoring information is limited in South America and particularly in Chile. Consequently, the chemical target list for micropollutant analysis in this study was primarily based on chemicals commonly detected in streams and rivers within European contexts. It is important to note that the classification of chemical classes is predominantly European/German-centric. The target screening encompassed 861 chemicals and was conducted employing an UltiMate 3000 LC system (Thermo Scientific, Germany) coupled with a quadrupole-Orbitrap MS (Q Exactive Plus, Thermo Scientific, Germany) featuring a heated electrospray ionization (ESI) source. A retrospective analysis, as outlined by Muschket et al. [7], was applied to 150 out of the 861 target chemicals. Furthermore, an additional evaluation was performed for the more hydrophobic analytes, 36 chemicals, utilising a TRACE 1310 GC system (Thermo Scientific, Germany) coupled with a quadrupole-Orbitrap MS (Q Exactive, Thermo Scientific, Germany) equipped with a Thermal Desorption Unit (TDU-2; Gerstel, Mülheim, Germany) and a cooled injection system (CIS; Gerstel).

ProteoWizard (version 2.1.0) was used to convert LC—HRMS raw data into mzML format [8]. Subsequently, peak detection, sample alignment, and target compound annotation were performed using MZmine (V 2.40.1) [9] as detailed elsewhere [10]. We used the R package {MZquant} (version 0.7.22) [11] to perform blank correction, calibration, and then quantification of the annotated target compounds. Blank peak elimination and blank intensity thresholds were calculated according to Machate et al., [6]. For the quantification of GC—HRMS detected compounds, the software TraceFinder 4.1 (Thermo Scientific) was used for further evaluation. Lastly, a series of method-matched calibration standards ranging from 0.5 to 5000 ng/L were used. The calibration standards were treated the same way as the river water samples. The target compounds were quantified using the internal standards with the nearest retention time following Nanusha et al. [5] and Machate et al. [6]. The method detection limits (MDLs) were determined following the US-EPA procedure [12].

### 3.3 Sample preparation and extraction procedure

LC–MS grade methanol, formic acid, and ammonium formate were procured from Honeywell, while LC–MS grade water was obtained from Thermo-Fisher. LC–MS grade ethyl acetate and 7 N ammonia in methanol were acquired from Sigma-Aldrich. GC-grade ethyl acetate was purchased from Merck (Darmstadt, Germany). Reference standards, with a purity exceeding 99%, were obtained from various suppliers. Isotopically labelled standards were purchased from Wellington Laboratories (Guelph, ON, Canada) and Campro Scientific (Berlin, Germany).

Preparation of the tailor-made solid phase extraction (SPE) cartridges involved preconditioning with methanol/ethyl acetate (1:1, v/v), methanol, and water prior to water sampling. In total, 50 litres of water were sampled, the cartridges were stored at 4 °C and transported to the laboratory. Upon arrival, the cartridges were purged with nitrogen to eliminate water, freeze-dried, and stored at −20 °C for extraction. Blank samples were prepared in a similar manner as the actual samples, utilising the same LVSPE device.

For sample preparation and extraction, we followed Machate et al. [6]. Finally, for LC analysis, 100-µL aliquots of the samples were spiked with 25 µL of an internal standard mixture comprising isotope-labelled compounds (1 µg/mL), along with 30 µL of methanol and 60 µL of water [5,6]. For GC analysis, 30 µl aliquots were taken and spiked with 1.6 µl GC-IS mix in order to have a final concentration of 50 ng/mL. All samples were measured with a method-matched calibration. The injection volume was set to 5 µl [5,6].

### 3.4 Liquid and gas chromatography HRMS

A Kinetex C18 EVO column (50 × 2.1 mm, 2.6 µm particle size), equipped with a pre-column (C18 EVO 5 × 2.1 mm) and an inline filter, was employed for LC separation. The gradient elution consisted of a mobile phase comprising 0.1% formic acid (eluent A) and methanol containing 0.1% formic acid (eluent B), used with a flow rate set at 300 µL/min. The elution protocol initiated with 5% eluent B for 1 min, followed by a linear increase in the fraction of eluent B to 100% over 12 min. Subsequently, 100% eluent B was maintained for 11 min. To remove hydrophobic matrix constituents from the column, a rinsing step was performed using a mixture of isopropanol and acetone (50:50) along with eluent B and eluent A (85%/10%/5%). The column was then re-equilibrated to the initial conditions for 5.7 min. An injection volume of 5 µL was used and the column was operated at 40 °C.

The heated electrospray ionization (ESI) source and transfer capillary were operated at 300 °C, with a spray voltage of 3.8 kV, a sheath gas flow rate of 45 arbitrary units (a.u.), and an auxiliary gas flow rate of 1 a.u. Full scan MS1 data was recorded in the mass-to-charge ratio (*m/z*) range of 100–1500, with a nominal resolving power of 140,000 (referenced to *m/z* 200) for metabolite confirmation. Additionally, data-dependent MS/MS acquisition was performed at a resolving power of 70,000 in separate runs. The MS instrument was externally calibrated every 2 days using calibration mixtures provided by the vendor, ensuring a mass accuracy within 5 ppm for all analyses. Both MS and MS/MS analyses were conducted in both positive (ESIpos) and negative (ESIneg) modes.

Gas chromatography separation was conducted using a DB-5MS capillary column (30 *m* × 250 µm, 0.25 µm film thickness; Agilent). Helium was used as the carrier gas at a constant flow rate of 1.2 mL/min. The oven program followed the protocol described by Muz et al. [13]. The transfer line temperature was maintained at 250 °C.

For injection, 2 µL of the extract aliquots were introduced into thermo-desorption tubes equipped with glass inserts, which acted as single-use liners. Upon injection, the thermal desorption unit was held at 80 °C for 4 min and then rapidly heated at a rate of 720 °C/min until reaching 300 °C. During the thermal desorption process, the sample was trapped in the cooled injection system at 10 °C, and subsequently, the injector temperature was increased at a rate of 720 °C/min until reaching 300 °C. Detailed information regarding the settings of the thermal desorption unit (TDU) and cooled injection system (CIS), as well as the GC oven program, can be found in the study by Muz et al. [13]. During high-resolution mass spectrometry (HRMS) measurements, the ion source temperature was set to 250 °C. Electron ionization was employed with an emission current of 50 µA and an electron energy of 70 eV. The mass spectrometer operated in full scan mode with a scan range of 70–810 *m/z*, at a resolving power of 60,000 (referenced to *m/z* 200).

## Limitations

The chemical target list for micropollutant analysis in this study was primarily based on chemicals commonly detected in streams and rivers within European contexts. Besides, the classification of chemical classes is predominantly European/German-centric.

## Ethics Statement

The proposed data does not involve any human subjects, animal experiments, or data collected from social media platforms.

## CRediT authorship contribution statement

**Pedro A． Inostroza:** Conceptualization, Methodology, Formal analysis, Investigation, Data curation, Writing – original draft, Writing – review & editing, Visualization. **Sebastian Elgueta:** Methodology, Investigation, Writing – review & editing. **Melis Muz:** Methodology, Validation, Formal analysis, Investigation, Writing – review & editing. **Martin Krauss:** Methodology, Validation, Investigation, Writing – review & editing. **Werner Brack:** Investigation, Resources, Writing – review & editing. **Thomas Backhaus:** Investigation, Resources, Writing – review & editing, Funding acquisition.

## Data Availability

Dataset of chemicals of emerging concern detected in streams and rivers of Central Chile (Original data) (zenodo).
